# A qualitative study of the infant feeding beliefs and behaviours of mothers with low educational attainment

**DOI:** 10.1186/s12887-016-0601-2

**Published:** 2016-05-21

**Authors:** Catherine Georgina Russell, Sarah Taki, Leva Azadi, Karen J. Campbell, Rachel Laws, Rosalind Elliott, Elizabeth Denney-Wilson

**Affiliations:** Faculty of Health, University of Technology Sydney, Sydney, Australia; Institute for Physical Activity and Nutrition, Deakin University, Melbourne, Australia

**Keywords:** Infant, Feeding behaviour, Pediatric obesity, Weight gain, Vulnerable populations, Mothers

## Abstract

**Background:**

Infancy is an important period for the promotion of healthy eating, diet and weight. However little is known about how best to engage caregivers of infants in healthy eating programs. This is particularly true for caregivers, infants and children from socioeconomically disadvantaged backgrounds who experience greater rates of overweight and obesity yet are more challenging to reach in health programs. Behaviour change interventions targeting parent-infant feeding interactions are more likely to be effective if assumptions about what needs to change for the target behaviours to occur are identified. As such we explored the precursors of key obesity promoting infant feeding practices in mothers with low educational attainment.

**Methods:**

One–on–one semi-structured telephone interviews were developed around the Capability Opportunity Motivation Behaviour (COM-B) framework and applied to parental feeding practices associated with infant excess or healthy weight gain. The target behaviours and their competing alternatives were (a) initiating breastfeeding/formula feeding, (b) prolonging breastfeeding/replacing breast milk with formula, (c) best practice formula preparation/sub-optimal formula preparation, (d) delaying the introduction of solid foods until around six months of age/introducing solids earlier than four months of age, and (e) introducing healthy first foods/introducing unhealthy first foods, and (f) feeding to appetite/use of non-nutritive (i.e., feeding for reasons other than hunger) feeding. The participants’ education level was used as the indicator of socioeconomic disadvantage. Two researchers independently undertook thematic analysis.

**Results:**

Participants were 29 mothers of infants aged 2–11 months. The COM-B elements of Social and Environmental Opportunity, Psychological Capability, and Reflective Motivation were the key elements identified as determinants of a mother’s likelihood to adopt the healthy target behaviours although the relative importance of each of the COM-B factors varied with each of the target feeding behaviours.

**Conclusions:**

Interventions targeting healthy infant feeding practices should be tailored to the unique factors that may influence mothers’ various feeding practices, taking into account motivational and social influences.

## Background

The increasing prevalence of childhood overweight and obesity globally [1–3] has led to a focus on strategies for their prevention and control [4–6]. In 2010 approximately 7 % (43 million) of children in United Nations regions aged 0–5 years were overweight or obese, up from approximately 4 % (27 million) in 1990. Furthermore another 14 % were at risk of becoming overweight [7]. Once established, overweight is difficult to treat [7, 8] and expensive [9], and many overweight infants remain overweight in childhood and beyond [8, 10]. Excess weight gain in infancy is a risk factor for overweight and obesity in later life [11] and is associated with numerous physical and psychosocial co-morbidities [12–14]. Importantly, the World Health Organization (WHO) now recognises infancy as an important focus for obesity prevention efforts [15]. The emphasis on the need to prevent obesity from the beginning of life acknowledges that alongside other important behaviours (i.e., sleep duration, sedentary and physical activity behaviours), diet, food preferences and eating behaviours are established in period of developmental plasticity and have longer-term health implications [16–18].

Despite our understanding of the importance of early life for obesity prevention relatively little is known about how best to engage and affect healthy eating, diet and weight in the early stages of life and until recently, this age group has been overlooked as a target for obesity prevention interventions [19, 20]. Although the determinants of child overweight and obesity are multifactorial [21, 22], for infants, the family context and interactions between infants and the primary caregiver, are significant [23]. Furthermore, to the extent that the behaviours and beliefs of the primary caregiver and the infant are considered malleable, these remain the likely most effective targets for obesity prevention efforts in infants and young children [24].

A particular challenge facing those developing family based obesity-prevention interventions is that the prevalence of overweight and obesity is socioeconomically patterned, with lower Socio-Economic Position (SEP) children being significantly more at risk than their higher SEP peers [25–27]. In Australia over one quarter (27 %) of Australian children from low SEP backgrounds are overweight or obese compared to approximately one fifth (19 %) of their more advantaged peers [28]. Given that socioeconomic inequalities in obesity begin in infancy [29, 30], efforts should be directed towards those approaches likely to be effective in lower SEP families. Important feeding practices that may explain such socioeconomic disparities in infant and child obesity incidence include (a) the use of infant formula instead of breast feeding [31–33] (b) feeding infants according to their appetitive cues instead of for other reasons; whether with infant formula, solid foods or breast milk (e.g., feeding to sooth, pressuring infants to finish all of the milk in the bottle) [34], (c) earlier age of introducing solid foods (before 4 months of age) as opposed to introduction of solid foods when the infant is approximately 6 months of age [17, 35] (d) suboptimal infant formula preparation (e.g., adding cereal to the bottle) [36] and (d) feeding young children unhealthy diets such as low levels of fruit and vegetable consumption in contrast to feeding children diets high health promoting foods like vegetables [37, 38].

Although these feeding practices have been identified as possible candidates for obesity prevention efforts, one challenge in addressing SEP differences in child overweight and obesity is that evidence-base upon which to design interventions with children and parents of low SEP backgrounds remains scant [4, 39]. That is, although socioeconomic patterning in obesity is well documented [3, 26], our mechanistic understanding of the reasons explaining this requires further exploration. Furthermore, when parents participate in obesity prevention programs there appears to be differential effects for parents of lower educational attainment and their children [40, 41] possibly due to the small knowledge base describing the determinants of healthy infant feeding practices in these groups. Given that the antecedents of feeding practices (e.g., beliefs, physical environments, social networks) are likely to differ with socio-demographic indicators such as ethnicity [42, 43] or SEP [44] they therefore require exploration in those groups in which the interventions are to be implemented. In the current study this was Australian mothers with low educational attainment.

**Fig. 1:**
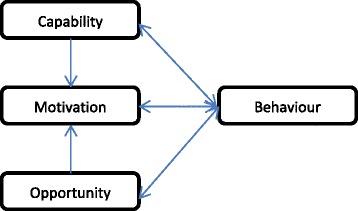
COM-B system showing interactions between elements of the framework (reproduced from Michie et al [50]

Michie’s Capability Opportunity Motivation Behaviour (COM-B) [45] framework, provides a structure in which to explore the determinants of health behaviours. This framework, illustrated in Fig. 1, represent the interactions between the different components of the behavioural system:

- the individual’s Capability (C), defined as a persons’ psychological or physical ability to enact the behaviour (e.g., knowledge, skills),
- the individual’s Opportunity (O), defined as the physical or social environment that enables the behaviour (e.g., availability of information, social support),
- and the individual’s Motivation (M), defined as the reflective (including self-conscious planning, analysis and decision-making) and the automatic (involving emotional reactions, drives, impulses and habits) mechanisms that may activate or inhibit behaviour [45].

The present study therefore explored, in a group of mothers with low educational attainment, the importance of the COM-B elements in affecting whether parents of low educational attainment adopt feeding practices associated with healthy, or excess, weight gain. It aimed to address existing gaps in knowledge about the antecedents of infant feeding practices in mothers of low educational attainment that could be used in the design of obesity prevention programs tailored to this high-risk group.

## Methods

### Study design

A qualitative study design was adopted to explore perceptions and behaviours of mothers about infant feeding practices by conducting one-on-one telephone interviews using a semi-structured interview guide. This approach was selected not only because it is an effective means of qualitative enquiry [46], but also because it allowed flexibility in interview times and locations, which we deemed essential in being able to reach a range of mothers with infants. Although telephone interviews have the disadvantage that visual cues (e.g., body language and facial expressions) are absent [47] it provides advantages of greater participant anonymity and cost effectiveness [47, 48]. Ethics approval was granted by University of Technology Sydney Human Research Ethics Committee (2013000463).

### Participants

Participants were recruited from two Australian regions (Australian Capital Territory, ACT and New South Wales, NSW). NSW and the ACT contain approximately a third of the total Australian population in both rural and urban settings. Mothers were eligible to participate if they had not completed a university degree (considered low educational attainment [49]; were the primary caregiver, were fluent in English, had an infant with no major health problems that may affect feeding, eating or growth (e.g., failure to thrive, chronic illness). The participants’ education level was used as the indicator of socioeconomic disadvantage as it has been shown, relative to other commonly used proxies for SEP (e.g., income or occupation) to be most strongly associated with the related concept of maternal diet [50] and has previously been used in our team’s research on feeding practices [51]. We targeted mothers with infants aged up to twelve months to allow us to capture the range of beliefs and behaviours associated with various infant feeding milestones.

### Recruitment

The study was advertised in the Playgroups NSW e-newsletter between January and March 2014. This newsletter is sent once a month to the 25,000 members of Playgroups NSW, a free program for parents and carers with children aged 0–5 years. Mothers who saw the advertisement in the newsletter subsequently shared the survey link with other mothers via social media, including a large Facebook group of mothers living in the ACT. The advertisement included a link to a web-based survey (Survey monkey®) where the interested mother provided demographic and contact details. These mothers were then screened according to their education level and age of their infant to assess their eligibility. Eligible participants were then sent a plain language participant letter and a consent form via e-mail. Mothers were asked to verbally consent to the study at the time of the interview and therefore no written or electronic consent form was completed.

### Interviews

The semi-structured interview guide was developed and structured in a way to enable us to address each of Michie et al’s COM-B framework components (Table 1). That is, we designed questions to explore the conditions that may affect each of the target behaviours. The target behaviours and their competing alternatives were informed by the literature as key behaviours related to obesity prevention in early life and included (a) initiating breastfeeding/formula feeding, (b) prolonging breastfeeding/replacing breast milk with formula, (c) introducing solids earlier than four months of age/delaying the introduction of solid foods until around 6 months of age, (d) feeding to appetite/use of non-nutritive (i.e., feeding for reasons other than hunger) feeding, and (e) introducing healthy first foods/introducing unhealthy first foods.

**Table 1:** Interview questions and prompts according to the target behaviours and Michie’s COM-B model

Target/Competing Feeding Behaviour	COM-B domain	Examples of interview questions/prompts
Initiating breastfeeding/Initiating formula feeding	Capability	Can you remember how you felt about the idea of breastfeeding when you were pregnant? Did you know much about breastfeeding?
	Opportunity	Did you receive any support or advice from anyone or anywhere about breastfeeding or formula feeding (Prompts: family, friends, media, antenatal education). If yes: What was the advice and support? Did it influence you?
		Are most of your friends breastfeeding or formula feeding?
	Motivation	When did you start thinking about whether you wanted to only breastfeed or formula feed him/her or do both? So you had/hadn’t planned on how you would feed your baby?
Prolonging breastfeeding/Introduce formula	Capability	Do you feel that you know how to breastfeed well now?
		Do you feel confident about it? Why/why not?
	Opportunity	Do you feel supported, practically or emotionally in (breast) feeding your baby?
		What things have influenced you to continue breastfeeding (Prompts: nutritional content, convenience, sleep better with BF or formula, work, friends).
	Motivation	Do you want to continue breastfeeding your baby?
		Are you still planning on breastfeeding for X?
Best practice formula preparation and feeding practices/Suboptimal formula preparation and feeding practices	Capability	How do you know how to make up the formula?
		Do you feel confident with formula feeding?
	Opportunity	Are there any issues around formula feeding that you would like more advice on or feel unclear about?
	Motivation	Which do you think is easier: Breast feeding or formula feeding?
Introducing solids later (at 6 months)/Introducing solids earlier (before 4 months)	Capability	How will you know when the timing is right?/How did you know when to introduce solid foods to your baby?
	Opportunity	Were you provided with any support or advice from anyone or anywhere about when to introduce solids foods to your baby? Did any of the advice/support change the age at which you introduced solid foods?
		What is normal within your social network- when do other mothers introduce solid foods? Has this influenced you?/Will this influence you?
	Motivation	Do you want to introduce solids when your baby is a particular age?
		What kinds of things influenced your plans? Probe: beliefs about the consequences of introducing solids at various ages.
		It is recommended that babies should start solids food at around 6 months of age. How do you feel about this recommendation?
Introduce healthy first foods/Introduce unhealthy first foods	Capability	Do you feel that you know enough about what you should feed your baby?
		How confident do you feel with feeding your baby now? Why/why not?
	Opportunity	Have you been provided with any specific support or advice from anyone or anywhere about what foods to feed your baby? (Prompt: who? what advice? what would help?). Did it influence what you feed your baby?
	Motivation	Is there anything in particular that you want your baby to eat?
		How confident do you feel with feeding your baby now?
Feed to appetite/Use non-nutritive feeding	Capability	What kinds of things influenced your (settling) behaviours? Probe: Knowledge, perceived ability.
		How do you know when to feed your baby? How do you know when your baby is hungry or full? How do you know how much to feed your baby?
	Opportunity	Were you provided with any support or advice from anyone or anywhere about settling your baby?
	Motivation	Do you find it [using milk or food to settle] effective?
		Before having your baby had you thought about what techniques you might use to settle the baby? (Prompt: Did you think that milk/food might be something that you would use?)
		Did you plan on stopping breastfeeding at a particular age? Do you want to stop breastfeeding your baby at that age?
		Which do you think is easier: Breast feeding or formula feeding?
		Is (stopping breastfeeding at a particular age) something you had planned on doing?

Interview guides were adapted according to the age and feeding milestones of the infant. For instance, mothers who had not yet introduced solids to their infants were not asked questions about their current solid food feeding behaviours but rather their intentions to introduce solid foods. The interview was piloted with 5 mothers meeting the same eligibility criteria as the main study. Refinements were made to the interview schedule to improve clarity and flow. Mothers were interviewed over the telephone by two of the investigators (ST and LA) at a time convenient to them. Interviews were audio recorded with participants’ permission. Mothers were offered an AUD30 supermarket voucher in appreciation of their time.

### Analysis

Interviews were transcribed verbatim and five randomly selected interviews were checked against the interview recording by ST to assess the accuracy of transcribing. Any sections of transcripts that were unclear were checked against the audio recordings. NVivo software [52] was used to code, store, sort and retrieve results from de-identified transcripts. Thematic analysis networks [53] was employed. Following Attride-Stirling [53], ST and CGR independently developed thematic coding manuals using the a priori selected theoretical model (COM-B) as a guide but being open to new codes emerging. In developing the manual, two iterations of coding took place with the two researchers each coding five transcripts to identify themes and relevant statements or quotes. Codes were organised into sub-themes and broader conceptual themes. The coding manual was revised and discussed after each iteration until both researchers were in agreement. These two investigators (ST and CGR) then independently coded all of the interviews. Any discrepancies in the coding manual and codes were resolved through discussion. The researchers used statistical measures of inter-coder verification using the Coding Comparison query in NVivo to identify the reliability of the study. This function calculates the percentage agreement between the two coders, which is the number of units of agreement divided by the total units of measure within the data item, presented as a percentage. Ten interviews were selected to conduct the coding comparison query including five from the interviews conducted with mothers that have not yet introduced solids and five from mothers that have introduce solids to their infant.

**Table 2:** Demographic profile of participants, their infants and the current feeding mode

	*N* (total = 29)
Participant characteristics	
Age (years) (M)	29 ± 8
Education	
Trade certificate	*n =* 17
Incomplete high school	*n =* 5
Complete high school	*n =* 6
Incomplete university degree	*n =* 1
Ethnicity (self-identified)	
Australian	*n =* 20
Caucasian	*n =* 5
Other	*n =* 4
Region	
NSW	*n =* 17
ACT	*n =* 12
Infant characteristics	
Age, months (M)	6.5 ± 4.5
Males	*n =* 16
Feeding mode	
Feeding solids	*n =* 18
Breastfeeding exclusively (in conjunction with solids)	*n =* 20 (*n =* 12)
Formula feeding exclusively (in conjunction with solids)	*n =* 7 (*n =* 5)
Mixed feeding exclusively (in conjunction with solids)	*n =* 2 (*n =* 1)
Older siblings	*n =* 14

## Results

Table 2 provides an overview of the participants’ characteristics. There were 120 mothers who expressed interest in participating. Of these, 29 mothers were eligible and were interviewed between February and March 2014. The mothers were aged 21–38 years, the majority self-identified as being of Australian background (*n =* 20), had completed trade certificates (*n =* 17), and came from NSW (*n =* 17). The infants were 13 girls and 16 boys, ranging in age from two to 11 months (M = 6.5 months). Most (*n =* 18) of the infants were eating solid foods, and were breastfed (*n =* 20). The interviews took on average 43 minutes (range 23–78 min) and data saturation was reached.

### Inter-rater reliability

Inter-rater reliability ranged from “poor” (Kappa <0.40) for the target behaviour *best practice formula feeding* (possibly due to the small number of participants who formula fed [54]) to “excellent” (Kappa >0.75) for the target behaviours *age of solids introduction* and *healthy first foods* with the remaining target behaviours being rated as “fair to good’ (Kappa 0.40 < 0.75) [52].

**Table 3:** Summary of the main themes and sub-themes arising from the interviews (*n =* 29)

	Main theme (COMB)	Sub Theme
Initiating breastfeeding/Initiating formula feeding		
Capability	- Physically establishing breastfeeding - Mental toughness	- The very first days are vital as it is so difficult for mothers - Having breastfed before means having more breastfeeding skills to get through the challenges with determination and strength
Opportunity	- Support and advice - Emotions - Social norms	- Whether the support and advice in hospital is adopted depends upon the individual (inconsistent) and whether nurses are pro-breastfeeding or accept formula feeding - Support from family and friends for choice of feeding mode, previous experience of breastfeeding, choosing to go own way (not influenced by others) - Others in social network are breastfeeding - Negative emotions associated with breastfeeding affect decision to shift to formula
Motivation	- Desire to breastfeed - Intentions/plans - Beliefs about the consequences (to baby) - Beliefs about the consequences (to mother) - Emotions	- There are benefits to the baby (nutrition and immunity) - Breastfeeding is good for bonding with the baby - It is convenient to breastfeed as no bottles are required - Breastfeeding can be very hard for the mother (e.g., mastitis) - Intentions/plans to breastfeed or formula made during pregnancy or earlier affect decisions about adopted feeding mode - Taking a pragmatic approach to feeding; willingness to use formula if necessary - Negative emotions (e.g., feelings of failure if unable to breastfeed, frustration with nurses, unable to cope with demands of breastfeeding) mean mother is likely to shift to formula feeding - Prior experience affected motivation (positive or negative)
Prolonging breastfeeding/Replacing breast milk with formula		
Capability	- Confidence in ability to continue - Knowledge about benefits to the baby	- Feel confident in knowing how to breastfeed well - Knowledge about health benefits to baby in continuing to breastfeed
Opportunity	- Work - Social norms	- It is too hard to express breast milk when going back to work - Social judgement and pressure to stop breastfeeding before the child is “too old”
Motivation	- Plans - Beliefs about benefits for baby - Wanting to do what is best for baby - Convenience/easier	- Plan to breastfeed for a minimum duration - Let the baby decide when he or she wants to stop (self-wean) - Baby has a preference for breastfeeding (does not take a bottle) - Baby’s characteristics affect whether breastfeeding is easy for the mother (e.g., baby pinches, gets teeth) - Breastfeeding is easy, convenient and cheap in comparison to formula
Best practice formula feeding/Suboptimal formula feeding practices		
Capability	- Confidence in ability to formula feed well	- Confidence in ability to formula feed well is high after an initial learning period
Opportunity	- Advice and support	- There is very little advice available from health professional so information provided on the formula tin is used - Some health professionals are judgemental towards mothers who formula feed and do not provide support - Social norms only influence some mothers
Motivation	- Motivated to feed well	- Mothers motivated to feed their infant well
Introducing solids later (at 6 months)/Introducing solids earlier (before 4 months)		
Capability	- Knowledge - Confidence	- It is confusing to know when is the best time to introduce solid foods - The baby gives cues and this is the best way to know - Mothers vary in their confidence about knowing when is the right time to introduce solids
Opportunity	- Advice - Social norms	- There is conflicting and confusing advice about when to introduce solids - Listen to advice but make up own mind about what is best for baby
Motivation	- Beliefs - Desires	- The 6 month government recommendation is not applicable to me and my baby (it is too broad, should be flexible, not tailored to individual needs) - The baby’s cues are the best indicator of when is the right time to introduce solid foods - Mothers know what is best for their babies - Introducing solids will have the benefit of improving baby’s sleep and alleviate hunger - There is no reason not to introduce solids early
Introduce healthy first foods/Introduce unhealthy first foods		
Capability	- Knowledge - Confidence	- Mother feels that she knows what foods baby should eat in relation to choking hazards, allergies and what is for good digestion - Mother’s confidence in knowledge of what foods to feed baby is affected by experience with solid food feeding, the baby’s weight and happiness, concerns about allergies and choking and whether she received confusing or clear advice - Mothers’ confidence is not necessarily related to her knowledge
Opportunity	- Advice	- Advice comes from health professionals, friends, family, online and it is inconsistent, confusing and often not practical - Good advice from a health professional is hard to come by - Advice affects mothers’ confidence - Advice online (blogs, Facebook etc.) is very helpful and practical. If mother cannot get good advice from health professionals she looks online - Mothers’ receptiveness to advice is varied with some mothers feeling they did not need advice
Motivation	- Desires/wants	- Heuristics help inform choice of foods (e.g., homemade food, fresh food, fruits & vegetables, unprocessed foods, no sugar or salt) - Mothers want to feed healthy foods, want to avoid allergenic foods - Want to give baby what s/he wants, take cue from the baby
Feed to appetite/Use non-nutritive feeding		
Capability	- Knowledge - Confidence	- Mother knows how to settle infant without milk/food - Feels confident that settling techniques work - Can accurately read baby’s cues (e.g., hunger or tiredness)
Opportunity	- Advice	- There is limited advice on settling techniques available to mothers - Advice from health professional is usually provided prior to birth and therefore is not timed with the mother’s need - Mothers seek information from multiple sources (e.g., nurse, family, books.) - There is very little advice available to mothers on how often and how much to feed infants
Motivation	- Beliefs about the consequences of the behaviour (efficacy) - Beliefs about baby’s needs	- Use whichever techniques work, try various options and see what works (process of deduction) - Feeding to settle works, but tend to use milk as a last resort for settling - There is nothing wrong with feeding to settle - Use the baby’s cues to determine whether to feed, trust the baby’s ability to know when hungry or full - Use a combination of the baby’s cues and the clock to determine whether to feed - Usually try and get the baby to eat/drink a set amount - Mothers usually hadn’t thought about or planned on how they might settle their infant before giving birth

A summary of the main findings is contained in Table 3 and a description of the findings for each of the target behaviours is provided below.

### Initiating breastfeeding/Initiating formula feeding

Initiation of breastfeeding or formula feeding began with a mother’s motivation (Reflective Motivation) to either breastfeed or formula feed [*She was going to be breastfed no matter what*. Breastfeeding mother 10]. This desire or plan often formed early – either in pregnancy or even before pregnancy. Some mothers had never considered an alternative to breastfeeding [“*I don’t think there was ever a time when I wasn’t going to breast feed*,” breastfeeding mother 1]. Reasons for planning to breastfeed were that it was broadly perceived as being nutritionally optimal for the infant [*Just because I knew it was good for her and I wanted to do wanted to do what was best for her and I wanted to do what my body is made for.* Breastfeeding mother 19], for bonding, health (e.g., immunity), ‘naturalness’, convenience and cost [*…just that’s what our breasts were made for so you may as well use them, and it’s free as well I guess, less hassle of doing bottles and having to spend extra money when you don’t have it.* Breastfeeding mother 2*].* However, other mothers were not motivated to breastfeed for reasons such as it feeling unnatural or strange [*Very uncomfortable. It’s strange, but yes, I definitely didn’t want to breastfeed at all. It definitely made me very uncomfortable and I didn’t breastfeed either of my children.* Formula feeding mother 2]. Finally, other mothers took a pragmatic approach whereby they planned to breastfeed but were aware that it ‘didn’t always work out’ *[Like I just wanted to try to be really relaxed, and if it worked it worked, and if it didn't I wasn't - like I was determined not to feel like a failure if I couldn't breastfeed.* Breastfeeding mother 3]. Mothers who had previously breastfed a baby were often more motivated to breastfeed (Reflective Motivation) and possessed more skills in breastfeeding (Physical Capability) as well as knowledge about how to breastfeed (Psychological Capability) *“I think being my second child breastfeeding, she’s just been very good at latching on and feeding since she was born, which is different to my first experience…the first few months, even though it was a lot easier”* Breastfeeding mother 21]. Whereas those who had previously had difficulties breastfeeding a child were the reverse *“it was my preference to breastfeed. But because I'd had trouble with my first baby…I was also a bit realistic in that it might not be an option for me. As it turned out, it wasn't an option for me” Breastfeeding mother 1].* Likewise, those who had a positive experience of formula feeding a previous infant (Behaviour) were also more likely to be Motivated to formula feed again *[Yeah, I never even considered breastfeeding with my second because I had such a good experience with bottle feeding with my first…so I decided to go the same way again.* Formula feeding mother 2].

For those mothers who were motivated to breastfeed, Physical Capability (breastfeeding skills) as well as Psychological Capability (mental toughness, determination) affected whether they took up breastfeeding after the baby was born. For example, this mother struggled with her infant’s reflux and weight loss and was advised that formula would help: [*But yes I mean I would have loved to give him all the benefits of the immune system and my health benefits and everything but it just wasn't suitable.* Formula feeding mother 3]. For instance for some mothers who had planned and wanted to breastfeed (Reflective Motivation), but experienced problems with latching or mastitis for instance (Physical Capability) this aroused negative emotions (Automatic Motivation) and reduced likelihood of them breastfeeding (Behaviour) [*after a month of breastfeeding I did give up after having mastitis three times and also suffering with post natal depression, it just wasn’t something that worked for me.* Formula feeding mother 5]. Mothers who were high in Mental Capability and/or were motivated (Reflective Motivation) were able to get through this difficult period and establish breastfeeding [*There was a stage where breastfeeding was hard and I was contemplating stopping, but I couldn’t bring myself to do it because I felt like it’s wrong to give him formula, like it’s not natural, like it’s a man-made thing and I want him to be as healthy and to grow up with the best possible start.* Formula feeding mother 3].

Opportunity was also important in affecting the initiation of breastfeeding or formula feeding: mothers who felt unsupported by hospital staff in breastfeeding (Social Opportunity) were more likely to lose motivation (Reflective Motivation) and experience more negative emotions (Automatic Motivation) which resulted in the competing behaviour being performed (introduction of infant formula) *[“the midwife was very - they didn't want to give any advice on formula feeding. Like they did push breastfeeding a lot which is fair but I don't think that - I think maybe if they didn't shame mothers so much with formula feeding there might be more mothers that mix fed” Breastfeeding mother 4].*

### Prolonging breastfeeding/Replace breast milk with formula

The main influences on the duration of breastfeeding appeared to be Reflective Motivation, relating to beliefs about the benefits of breastfeeding to the baby and to the mother (convenience and ease) as well as mothers’ plans or goals to achieve a minimum duration of breastfeeding *[Your body’s got everything that your baby needs… there are so many different types of bacteria and stuff in breast milk…But we can’t make those in the formula…, I can just go out with my baby and just stop and breastfeed for a second and all these sorts of things, yeah, whereas getting bottles and formula and stuff like that, it does cost a lot of money and it’s good. Breastfeeding mother 7].* Social Opportunity (norms) seemed to have less of an influence on this behaviour. Representing an Environmental Opportunity barrier, returning to work was often the impetus for stopping breastfeeding *[…I’m going back to work when he’s nine months old so I’ll probably feed him until probably seven months, so I can get him on the bottle before I go back to work.* Formula feeding mother 2]. The influence of the infant on the mother was also important. Mothers were physically unable to continue breastfeeding (Physical Capability) when their infant self-weaned *[She’s pretty much self-weaning, she’s not really interested.* Mixed feeding mother 1], whilst others were motivated (Reflective Motivation) to continue breastfeeding because their infant did not like to drink milk from a bottle *[I don't have a choice, they don't like bottles. Breastfeeding mother 11].* Advice (Environmental Opportunity) did not appear to have an influence on breastfeeding duration in that advice on breastfeeding appeared to be given to mothers only during pregnancy or just after birth.

### Best practice formula preparation/Sub-optimal formula preparation

Social Opportunity and Environmental Opportunity were barriers towards best practice formula feeding: Mothers mentioned there was little support and information available to those who formula feed their infant. Furthermore, mothers reported that they felt judged and unsupported by health professionals who were perceived to be pro-breastfeeding *[I mean, when you first have a bub you’re thrown into, I suppose, breastfeeding and you’re given so much advice and so much support based on that, but if you have to change to formula or something like that, it’s very negatively viewed upon, even by health practitioners.* Formula feeding mother 5]*.* For this reason advice and information on how to formula feed came primarily from the formula tin, and through online searches. Most mothers appeared to avoid putting their infant to bed with a bottle of formula and routinely followed instructions on tin about formula preparation, adjusting the volume of formula to their infant’s hunger levels *[No. I mean I was breastfeeding for the first 6 months, so I used the Australian Breastfeeding line for advice on that. With the formula, I just go off the instructions off the formula bottles and off her cues as well.* Formula feeding mother 1]. Mothers were confident in their ability to formula feed their infants (Psychological Capability). Whether others were formula feeding in their social network didn't appear to have much influence.

### Introducing solids later (at 6 months)/Introducing solids earlier (before 4 months)

Reflective Motivation emerged as the main barrier towards introducing solids in line with Australian Infant Feeding Guidelines [55]: Participants rarely mentioned a desire to wait until their infant was six months of age before introducing solid foods *[No, I don’t think it’s [waiting until 6 months] realistic at all. Every baby’s different and if we had of waited for her to be six months, she wouldn’t have been very happy at all. Formula feeding mother 2].* This is a rare quote from a mother who was in favour of waiting: *“yeah I think so…other mum’s will say I had to start earlier because they were looking at my food and wanting to put it in their mouth and I sort of think, well, babies look at everything and want to put it in your mouth” Breastfeeding mother 5].* In contrast, Reflective Motivation to engage in the competing behaviour (introducing solids early) was higher. The reasons were related to mothers’ beliefs about the consequences of the behaviour (it was perceived as beneficial to the infant to introduce solids early, for example reducing hunger, sleeping longer) [*I don't think it's realistic [waiting until 6 months], because if a baby shows that they're ready, I think just go with what your baby's telling you. Because instead of being like them wanting more and more feeds - it's breaking their sleep as well, and they're not getting any sleep.* Breastfeeding mother 6*].* Mothers mentioned several potential benefits of introducing solid foods earlier, yet appeared to have few beliefs about possible negative impacts to their infants of introducing solid foods earlier, and affected the age at which solid foods were introduced. Mothers were also motivated (Reflective Motivation) to introduce solids when they perceived their baby to be ready (indicated by signs/cues), rather than based on health recommendations *[I think it’s [government recommendation] open to interpretation in the fact that okay, each parent knows their child best and every child develops differently. And if some children need to have solids earlier, then who’s to say that they can't?* Formula feeding mother 7]. Furthermore, although most mothers appeared knowledgeable about the recommended age at which solids should be introduced some mothers were confused (Psychological Capability) about when to introduce solids *[I guess the information on when to start solids is probably more confusing than the breastfeeding information almost. It's like - because it does seem to change a bit but - and then I've heard that if you start them - the earlier you start them the less likely they are to have allergies but then I don't know whether that's true or not. Breastfeeding mother 8].* Social pressure or social norms (Social Opportunity) was also important with mothers recounting receiving pressure or advice from family members or peers to introduce solids at early ages which some mothers chose to ignore *[…my mother-in-law suggested that I start giving him solids at two months, so I think that’s the older way of going about doing things which I absolutely refused to do. Formula feeding mother 5].*

### Introduce healthy first foods/Introduce unhealthy first foods

Not surprisingly, mothers were Motivated (Reflective and Automatic motivation) to give those foods that they believed was best for their baby (what their baby needed or wanted) *[I look at the way she is. I started her on purees and I could tell that she wasn’t interested in her food anymore, so I tried something different, like mashed food. Now she’s a bit over it, so I’m trying finger food.* Breastfeeding mother 9]. Mothers reported having made plans about which foods they would like to introduce and which they would like to avoid, often relying upon heuristics such as ‘fresh foods’, ‘no packaged foods’, ‘no sugar’, ‘homemade’, ‘fruits and vegetables’ [*I guess I've always thought fresh is best. So I always try where I can to give him fresh food, wholesome food. Formula feeding mother 6].* Mothers were also motivated to avoid allergenic foods and those that may pose a choking hazard *[I've heard that if you start them - the earlier you start them the less likely they are to have allergies but then I don't know whether that's true or not. Breastfeeding mother 4].* Confidence (Psychological Capability) was affected by past Behaviour (having had a child previously) their infant’s reactions (e.g., eating the food, gaining weight) and further experience of feeding their infant (more time after introducing solids). Confidence, as well as knowledge (Psychological Capability) was negatively affected by receiving confusing advice (Opportunity) about which foods to give to infants at different stages of development and with inexperience (early on in the introduction of solids period) *[This is where I get confused as well, because people say you need to start on fruit first. Some people say Farex, and other people say vegetables. Breastfeeding mother 3].* For example, mothers relied upon online blogs, popular books, Google searches and Facebook, or on family/friends as a source of information about which foods to give infants in the absence of other reliable and timely information from health professionals *[No, only on Facebook group that the mums were talking about what they were going to be introducing to their kids. But other than that, just a hundred percent reliant upon the book really. I kind of take what the Facebook group says with a bit of a grain of salt sometimes Formula feeding mother 4]*.

### Feed to appetite/Use non-nutritive feeding

Mothers’ desires (Reflective Motivation) to use non-nutritive feeding (primarily feeding to settle) appeared to be higher than desires to feed according to their infant’s appetitive cues. A desire, plan or perceived need to avoid non-nutritive feeding was absent (Reflective Motivation). Mothers often said that they would do ‘whatever works’ to settle the baby and this often included offering milk to their infant. Furthermore, mothers’ beliefs about the consequences of using milk to settle were positive, as it was perceived as an effective settling technique (Reflective Motivation). There was very little indication that using milk to settle the infant would have any negative consequences for the infant (Reflective Motivation). *[He will just follow me around, like he crawls, just crying at me until I give him a biscuit or a bottle. Then he's fine, as long as he's like been given something he's happy.* Formula feeding mother 2]. Social Opportunity was also a barrier towards mothers’ use of feeding to appetite: Mothers recounted that although at times they were given advice on how to settle their infant without milk from health professionals, family or peers, they were given little support or advice on or information about the possible negative consequences for the infant of using non-nutritive feeding *[…we were told that we were doing the wrong thing with (baby’s name) by cuddling or feeding her to sleep. Breastfeeding mother 3].* However, other aspects of Motivation, such as making plans about feeding to appetite or to settle were largely absent from the discussion about feeding to appetite/use of non-nutritive feeding.

Aside from using milk/food to settle the infant, many mothers did report allowing their infant to stop feeding when full (Behaviour) *[No I just purely go on if she's eating it, like I go on her cues. If she's full, if she's not interested, then that's enough. Sometimes she might not even eat any of it.* Formula feeding mother 1]. This was largely affected by Reflective Motivation: mothers believed that the infant was able to determine if she or he has full *[He'll pull off the bottle, his head will turn to the side and he just won't latch back on so we just don't - we offer it to him. If he doesn’t want it - he knows his own body more than we do.* Formula feeding mother 3], although there were also mothers who tried to get their infants to eat a certain amount of food/milk, believing that the infant required more food than he/she wanted [*Facilitator: Yeah, that’s right. So with the formula, is there a particular amount that you do try to give to him every day? Interviewee: Anything over the 500 mark, as long as he’s having roughly 500mls a day.* Formula feeding mother 7].

## Discussion

In the present study we explored, amongst mothers with low educational attainment, key factors influencing those infant feeding practices shown to be important in influencing excess weight gain. The factors affecting mothers’ use of the feeding practices could be mapped to each of Michie et al’s COM-B elements [45]. Interestingly, each of the COM-B factors varied in their importance as a determinant of the target feeding behaviours, thus providing insights into targeted strategies required in healthy feeding interventions.

Overall, the COM-B elements of Social and Environmental Opportunity, Psychological Capability, and Reflective Motivation were the key elements identified as determinants of a mother’s likelihood to adopt the healthy target behaviours. Importantly, though, the results showed that their significance varied for each of the target behaviours. For instance, for some target behaviours (e.g., age of introducing solids) mothers appeared to have the necessary knowledge or skills to perform the behaviour (Capability), however their Reflective Motivation (e.g., beliefs about the consequences of performing the behaviour) was the factor that appeared more likely to affect their decision to introduce solids earlier rather than later. In contrast, for other target behaviours (e.g., introducing healthy first foods) Capability (e.g., knowledge) and Opportunity (e.g., advice) were most important. For these target behaviours, mothers were motivated (Motivation) to perform the target behaviour yet were unsure which foods could be safely offered at what age. Enacting the Behaviour earlier (e.g., with an older sibling, or for several months with their current infant) also influenced skills, confidence (Capability) and Motivation in some instances. This is expected in the COM-B model as shown by the directions of the arrows (Fig. 1).

A common theme across all of the target behaviours was the influence of the COM-B element Social and Environmental Opportunity, which included norms, advice and prompts from peers, health professionals and family members. For some of the target behaviours (e.g., initiation of breastfeeding) there appeared to be ample advice offered to mothers from credible sources, whereas for others (e.g., best practice formula feeding) advice was lacking. Seeking advice and support online was a common practice, with mothers relying upon social networking sites, government and health websites or commercial providers, which vary considerably in their quality [56]. Consistent with earlier findings [57, 58] mothers were, at times, dismissive of the advice and support provided by health professionals because it was not seen as practical or not relevant to their baby’s specific needs. Previously, other studies [58, 59] have shown that mothers consider advice from family and friends to be of more value than that from health professionals. One reason for this may be that mothers are seeking practical advice on infant feeding that can help to solve a perceived problem and will reject professional advice if it does not work for their infant [58]. Despite this, several mothers in this study also reported not following the advice from family members of older generations (mothers and mother in-laws) believing it was out-dated advice, which was similar to another study [60]. Therefore, given that mothers are influenced by advice from various sources, the credibility of the source and the accuracy of the advice remains a concern.

Motivation was an important influence on all of the target/competing behaviours*.* In the present study, Motivation referred to both Automatic (e.g., emotions) and Reflective Motivation. However it was Reflective Motivation that appeared to have the widest ranging influence on the target behaviours. Important elements of Reflective Motivation were the mothers’ beliefs about the consequences of their behaviours as well as the plans they made. The consequences of performing the target behaviour were at times perceived as negative, while the competing behaviour were positive. For instance, for the *feeding to appetite* and *introducing solids* target behaviours, mothers were more motivated to perform the competing behaviours (using milk to settle, introducing solids before 6 months) at least partly because they did not perceive any potential negative consequences of doing so. In fact mothers have been shown to prefer infants who eat a lot, are full and satisfied [61] and heavier [62] and some of the target feeding behaviours may be perceived to be in competition with this. Others have also noted that parents’ beliefs about the consequences of their behaviours are a barrier towards healthy feeding practices [59] and that parents’ knowledge about the effects of their feeding behaviours on their infants mediate obesity prevention intervention effects [63] and directly affect their choice of feeding or settling behaviour [64]. Therefore, mothers’ beliefs about the consequences of performing the target behaviour or its competing alternative directly affect their decision as to which behaviour to undertake.

Mothers’ Motivations were also affected by their infant’s characteristics and behaviours and the mother’s perceptions of them. Mothers made judgements about what their infant needed and took feedback from their baby’s cues (e.g., behaviours, sounds, growth) (Reflective Motivation), usually in combination with their knowledge or beliefs about what was best for the baby (Capability). Thus, at times, the Motivation to do the competing behaviour (e.g., feed to settle) was higher than the target behaviour (e.g., feed to appetite). Models of non-responsive feeding [65] suggest that parents who are less responsive to their infant’s hunger and satiety cues use feeding practices such as pressure, coercion and restriction, which are associated with excess weight gain [66]. Our results suggest a possible role for parents’ motivations in determining whether parents use these non-responsive feeding practices.

The information provided here allowed us to identify what is required for the healthy feeding practices to occur. As such, the next step is to link these to evidence-based strategies for changing them. According to Michie et al. [45] interventions will be effective if they change one or more of the COM-B components using the evidence-based Behaviour Change Techniques (BCTs) as specified in the Behaviour Change Wheel (BCW) framework of intervention design. While knowledge is identified as a key a barrier to healthy feeding practices [67], motivational and external (e.g., social, health professionals) influences are also important intervention targets. The data presented in this study suggest that for mothers to practice healthy feeding practices, two key areas could be targeted, with tailoring to the specific target behaviours of interest. These areas are (a) Social Opportunity and (b) Reflective Motivation.

An important finding of this study was the lack of reliable, timely, practical advice tailored to existing parent motives and varying child characteristics that is framed in motivating ways. As such, Social Opportunity was a barrier to healthy feeding practices when advice given by health professionals was ignored, not sought out or not optimised and when other social influences (e.g., peers, friends) provided social norms and advice contradictory to healthy infant feeding guidelines. Michie et al. [68] state that Social Opportunity may be influenced by environmental restructuring (e.g., providing prompts or cues), modelling (e.g., demonstrating the behaviour) or enablement (e.g., behavioural support from health professionals). However, for this advice to be taken up by mothers it must be delivered by a source perceived to be trustworthy, experienced and empathetic [58]. According to the COM-B model, Opportunity may influence Motivation and Behaviour yet not Capability. However, the present results suggested that Opportunity in the form of advice and support is likely to affect knowledge (Capability). For example, advice from health professionals or family members about how to breastfeed or about government recommendations on when to introduce solid foods affects parents’ knowledge on these topics.

As noted earlier, Reflective Motivation was also an important influence on several of the target behaviours. This could be targeted via Capability and Opportunity (see Fig. 1). The use of advice/support (Opportunity), for example, skill building (Capability) or expansion of knowledge (Capability) may help to change motivation [45]. For instance, mothers’ positive beliefs about the consequences of some behaviours (e.g., feeding to settle) act as a barrier towards feeding to appetite, and may be difficult to change in interventions unless a competing behaviour (using other behaviours to settle) is more effective and/or the mothers’ understanding of the longer term consequences of performing this behaviour is high. This behaviour may therefore be targeted by increasing mothers’ knowledge about the longer term consequences of using milk or food to settle infants (e.g., disruption of satiety responsiveness), providing mothers with greater skills in settling without food or milk and by emphasising the positive outcomes for both the mother and child in promoting the adoption of healthy feeding practices. This approach may be successful as there is already evidence that mothers are interested in and receptive to advice on ways to calm a fussy infant [69, 70].

This study provided novel insights into factors influencing infant feeding behaviours used by mothers with low educational attainment. However, the results should be viewed in the context of the study’s limitations. We used mothers’ education level as an indicator of low SEP although there may be differences between these and other disadvantaged mothers (e.g., those of low income, low occupational prestige) and the results may therefore not be generalisable to other disadvantaged groups. As we did not collect data on income we are unable to determine whether these mothers could be classified as being from low income households. Similarly, our sample predominantly identified as Australian or Caucasian and feeding beliefs and behaviours may differ with ethnicity. Our study was also limited by a small number of mothers who formula fed their infants, which was reflected in the relatively low kappa score for the coding of this target behaviour and further explorations of feeding practices in this group are needed. These data provide new information from a high risk but under researched group and this study is amongst the first to attempt to apply the COM-B framework to barriers and facilitators to healthy infant feeding practices in low SEP mothers, which adds to body of work on how to promote healthy feeding in this high-risk group.

## Conclusion

We investigated the factors affecting infant feeding practices linked with the onset of obesity in infancy in a group of low SEP mothers within the COM-B framework. Despite some commonalities across the target behaviours, especially with regards to the important influence of Social Opportunity, Psychological Capability and Reflective Motivation, it was also clear that the relative importance of each of the COM-B elements differed with each of the target behaviours. Furthermore, mothers’ receptiveness to health promotion interventions may depend upon child’s characteristics as well as mother’s characteristics and extant beliefs, experience with other child and access to support networks. Interventions targeting healthy infant feeding practices should therefore be tailored to the unique factors that may influence mothers’ various feeding practices, taking into account motivational and social influences.

### Availability of data and materials

The data supporting the findings are contained within the manuscript and tables.

## Abbreviations

ACT: Australian Capital Territory
COM-B: Capability, Opportunity, Motivation Behaviour framework
NSW: New South Wales
SEP: Socio-Economic Position
WHO: World Health Organization

## References

[1] Wang Y, Lobstein T (2006). Worldwide trends in childhood overweight and obesity. Int J Pediatr Obes.

[2] de Onis M, Blossner M, Borghi E (2010). Global prevalence and trends of overweight and obesity among preschool children. Am J Clin Nutr.

[3] Wake M, Hardy P, Canterford L, Sawyer M, Carlin J (2007). Overweight, obesity and girth of Australian preschoolers: Prevalence and socio-economic correlates. Int J Obes (Lond).

[4] Laws R, Campbell K, van der Pligt P, Russell G, Ball K, Lynch J, Crawford D, Taylor R, Askew D, Denney-Wilson E (2014). The impact of interventions to prevent obesity or improve obesity related behaviours in children (0–5 years) from socioeconomically disadvantaged and/or indigenous families: a systematic review. BMC Public Health.

[5] Skouteris H, McCabe M, Swinburn B, Newgreen V, Sacher P, Chadwick P (2011). Parental influence and obesity prevention in pre-schoolers: a systematic review of interventions. Obes Rev.

[6] Hung LS, Tidwell DK, Hall ME, Lee ML, Briley CA, Hunt BP (2015). A meta-analysis of school-based obesity prevention programs demonstrates limited efficacy of decreasing childhood obesity. Nutr Res.

[7] de Onis M, Lobstein T (2010). Defining obesity risk status in the general childhood population: which cut-offs should we use?. Int J Pediatr Obes.

[8] Stettler N, Iotova V (2010). Early growth patterns and long-term obesity risk. Curr Opin Clin Nutr Metab Care.

[9] Pelone F, Specchia ML, Veneziano MA, Capizzi S, Bucci S, Mancuso A, Ricciardi W, de Belvis A (2012). Economic impact of childhood obesity on health systems: a systematic review. Obes Rev.

[10] Hesketh K, Wake M, Waters E, Carlin J, Crawford D (2004). Stability of body mass index in Australian children: a prospective cohort study across the middle childhood years. Public Health Nutr.

[11] Weng SF, Redsell SA, Swift JA, Yang M, Glazebrook CP (2012). Systematic review and meta-analyses of risk factors for childhood overweight identifiable during infancy. Arch Dis Child.

[12] Huxley R, Shiell A, Law C (2000). The role of size at birth and postnatal catch-up growth in determining systolic blood pressure: a systematic review of the literature. J Hypertens.

[13] Law C, Shiell A, Newsome C, Syddall H, Shinebourne E, Fayers P, Martyn C, de Swiet M (2002). Fetal, infant, and childhood growth and adult blood pressure: a longitudinal study from birth to 22 years of age. Circulation.

[14] Parker L, Lamont D, Unwin N, Pearce M, Bennett S, Dickinson H, White M, Mathers J, Alberti K, Craft A (2003). A lifecourse study of risk for hyperinsulinaemia, dyslipidaemia and obesity (the central metabolic syndrome) at age 49–51 years. Diabet Med.

[15] World Health Organisation (2014). Report of the first meeting of the Ad hoc working group on science and evidence for ending childhood obesity.

[16] Ong KK (2006). Size at birth, postnatal growth and risk of obesity. Horm Res Paediatr.

[17] Kramer MS (2010). Breastfeeding, complementary (solid) foods, and long-term risk of obesity. Am J Clin Nutr.

[18] Gluckman P, Hanson M (2008). Developmental and epigenetic pathways to obesity: an evolutionary-developmental perspective. Int J Obes (Lond).

[19] Paul I, Williams J, Anzman-Frasca S, Beiler J, Makova K, Marini M, Hess L, Rzucidlo S, Verdiglione N, Mindell J (2014). The Intervention Nurses Start Infants Growing on Healthy Trajectories (INSIGHT) study. BMC Pediatr.

[20] Hesketh KD, Campbell KJ (2010). Interventions to prevent obesity in 0–5 year olds: An updated systematic review of the literature. Obesity.

[21] Davison K, Birch L (2001). Childhood overweight: a contextual model and recommendations for future research. Obes Rev.

[22] Nader PR, Huang TT, Gahagan S, Kumanyika S, Hammond RA, Christoffel KK (2012). Next steps in obesity prevention: altering early life systems to support healthy parents, infants, and toddlers. Child.

[23] Strauss RS, Knight J (1999). Influence of the home environment on the development of obesity in children. Pediatrics.

[24] Thompson AL, Adair LS, Bentley ME (2013). Pressuring and restrictive feeding styles influence infant feeding and size among a low-income African-American sample. Obesity.

[25] King T, Kavanagh AM, Jolley D, Turrell G, Crawford D (2006). Weight and place: a multilevel cross-sectional survey of area-level social disadvantage and overweight/obesity in Australia. Int J Obes (Lond).

[26] Wang Y, Beydoun MA (2007). The obesity epidemic in the United States--gender, age, socioeconomic, racial/ethnic, and geographic characteristics: a systematic review and meta-regression analysis. Epidemiol Rev.

[27] Shrewsbury V, Wardle J (2008). Socioeconomic status and adiposity in childhood: a systematic review of cross-sectional studies 1990–2005. Obesity.

[28] Australian Bureau of Statistics (2013). Australian health survey: updated results, 2011–2012.

[29] Wijlaars LPMM, Johnson L, van Jaarsveld CHM, Wardle J (2011). Socioeconomic status and weight gain in early infancy. Int J Obes (Lond).

[30] Gibbs BG, Forste R. Socioeconomic status, infant feeding practices and early childhood obesity. Pediatr Obes. 2013. 10.1111/j.2047-6310.2013.00155.x 23554385

[31] Dewey K, Heinig M, Nommsen L, Peerson J, Lonnerdal B (1993). Breast-fed infants are leaner than formula-fed infants at 1 y of age: the DARLING study. Am J Clin Nutr.

[32] Sherburne Hawkins S, Law C (2006). A review of risk factors for overweight in preschool children: a policy perspective. Int J Pediatr Obes.

[33] Dewey KG, Heinig MJ, Nommsen-Rivers LA (1995). Differences in morbidity between breast-fed and formula-fed infants. J Pediatr.

[34] Paul I, Bartok C, Downs D, Stifter C, Ventura A, Birch L (2009). Opportunities for the primary prevention of obesity during infancy. Adv Pediatr.

[35] Schack-Nielsen L, Sorensen T, Mortensen EL, Michaelsen KF (2010). Late introduction of complementary feeding, rather than duration of breastfeeding, may protect against adult overweight. Am J Clin Nutr.

[36] Lilburne A, Oates R, Thompson S, Tong L (1988). Infant feeding in Sydney: a survey of mothers who bottle feed. J Paediatr Child Health.

[37] Boumtje PI, Huang CL, Lee J-Y, Lin B-H (2005). Dietary habits, demographics, and the development of overweight and obesity among children in the United States. Food Policy.

[38] Pearce J, Langley-Evans SC (2013). The types of food introduced during complementary feeding and risk of childhood obesity: A systematic review. Int J Obes (Lond).

[39] Russell CG, Taki S, Laws R, Azadi L, Campbell KJ, Elliott R, Lynch J, Ball K, Taylor R, Denney-Wilson E (2016). Effects of parent and child behaviours on overweight and obesity in infants and young children from disadvantaged backgrounds: systematic review with narrative synthesis. BMC Public Health.

[40] Cameron AJ, Ball K, Hesketh KD, McNaughton SA, Salmon J, Crawford DA, Lioret S, Campbell KJ (2014). Variation in outcomes of the Melbourne Infant, Feeding, Activity and Nutrition Trial (InFANT) Program according to maternal education and age. Prev Med.

[41] Beauchamp A, Backholer K, Magliano D, Peeters A (2014). The effect of obesity prevention interventions according to socioeconomic position: a systematic review. Obes Rev.

[42] Cartagena DC, Ameringer SW, McGrath J, Jallo N, Masho SW, Myers BJ (2014). Factors contributing to infant overfeeding with Hispanic mothers. J Obstet Gynecol Neonatal Nurs.

[43] Anderson CB, Hughes SO, Fisher JO, Nicklas TA (2005). Cross-cultural equivalence of feeding beliefs and practices: The psychometric properties of the child feeding questionnaire among Blacks and Hispanics. Prev Med.

[44] Baughcum AE, Powers SW, Johnson SB, Chamberlin LA, Deeks CM, Jain A, Whitaker RC (2001). Maternal feeding practices and beliefs and their relationships to overweight in early childhood. J Dev Behav Pediatr.

[45] Michie S, van Stralen MM, West R (2011). The behaviour change wheel: a new method for characterising and designing behaviour change interventions. Implement Sci.

[46] Ritchie J, Lewis J, Nicholls CM, Ormston R (2013). Qualitative research practice: A guide for social science students and researchers: Sage.

[47] Novick G (2008). Is there a bias against telephone interviews in qualitative research?. Res Nurs Health.

[48] Neuman WL (2005). Social research methods: Quantitative and qualitative approaches, vol. 13.

[49] Zarnowiecki D, Ball K, Parletta N, Dollman J (2014). Describing socioeconomic gradients in children’s diets – does the socioeconomic indicator used matter?. Int J Behav Nutr Phys Act.

[50] Zarnowiecki DM, Dollman J, Parletta N (2014). Associations between predictors of children's dietary intake and socioeconomic position: a systematic review of the literature. Obes Rev.

[51] Campbell K, Hesketh K, Crawford D, Salmon J, Ball K, McCallum Z (2008). The infant feeding activity and nutrition trial (INFANT) an early intervention to prevent childhood obesity: cluster-randomised controlled trial. BMC Public Health.

[52] QSR International Pty Ltd (2012). NVivo qualitative data analysis software.

[53] Attride-Stirling J (2001). Thematic networks: an analytic tool for qualitative research. Qual Res.

[54] Banerjee M, Capozzoli M, McSweeney L, Sinha D. Beyond kappa: A review of interrater agreement measures. Can J Stat/La Revue Canadienne de Statistique. 1999;3–23.

[55] National Health and Medical Research Council (2012). Infant feeding guidelines.

[56] Taki S, Campbell KJ, Russell CG, Elliott R, Laws R, Denney-Wilson E (2016). Infant feeding websites and apps: a systematic assessment of quality and content. Interact J Med Res.

[57] Karp SM, Lutenbacher M, Dietrich MS (2010). The associations of psychosocial factors and infant feeding beliefs and practices of young, first time, low income mothers. Issues Compr Pediatr Nurs.

[58] Heinig MJ, Ishii KD, Banuelos JL, Campbell E, O'Loughlin C, Vera Becerra LE (2009). Sources and acceptance of infant-feeding advice among low-income women. J Hum Lact.

[59] Horodynski M, Olson B, Arndt MJ, Brophy-Herb H, Shirer K, Shemanski R (2007). Low-income Mothers’ decisions regarding when and Why to introduce solid foods to their infants: influencing factors. J Community Health Nurs.

[60] Redsell SA, Atkinson P, Nathan D, Siriwardena AN, Swift JA, Glazebrook C (2010). Parents beliefs about appropriate infant size, growth and feeding behaviour: implications for the prevention of childhood obesity. BMC Public Health.

[61] Heinig MJ, Follett JR, Ishii KD, Kavanagh-Prochaska K, Cohen R, Panchula J (2006). Barriers to compliance with infant-feeding recommendations among low-income women. J Hum Lact.

[62] Baughcum AE, Burklow KA, Deeks CM, Powers SW, Whitaker RC (1998). Maternal feeding practices and childhood obesity: a focus group study of low-income mothers. Arch Pediatr Adolesc Med.

[63] Spence A, Campbell K, Crawford D, McNaughton S, Hesketh K (2014). Mediators of improved child diet quality following a health promotion intervention: the Melbourne InFANT Program. Int J Behav Nutr Phys Act.

[64] Boyington JA, Johnson AA (2004). Maternal perception of body size as a determinant of infant adiposity in an African-American community. J Natl Med Assoc.

[65] Satter E (2000). Child of mine: feeding with love and good sense.

[66] Savage J, Fisher J, Birch L (2007). Parental influence on eating behavior: conception to adolescence. J Law Med Ethics.

[67] Kavanagh KF, Habibi M, Anderson K, Spence M (2010). Caregiver- vs infant-oriented feeding: a model of infant-feeding strategies among special supplemental nutrition program for women, infants, and children participants in rural east Tennessee. J Am Diet Assoc.

[68] Michie S, Johnston M, Francis J, Hardeman W, Eccles M (2008). From theory to intervention: mapping theoretically derived behavioural determinants to behaviour change techniques. Appl Psychol.

[69] Paul I, Savage J, Anzman S, Beiler J, Marini M, Stokes J, Birch L (2011). Preventing obesity during infancy: a pilot study. Obesity (Silver Spring).

[70] Kavanagh KF, Cohen RJ, Heinig MJ, Dewey KG (2008). Educational intervention to modify bottle-feeding behaviors among formula-feeding mothers in the WIC program: impact on infant formula intake and weight gain. J Nutr Educ Behav.

